# Minutes of the Proceedings of the Buffalo Med. Association

**Published:** 1854-12

**Authors:** Sanford B. Hunt

**Affiliations:** Sec’y


ART. IV.—*Minutes of the Proceedings of the Buffalo Medical Association at its Meeting held at the office of Dr. Wyckoff, on Tuesday, Nov.* 7, 1854. Reported by the SECRETARY.

After the transaction of the preliminary business, Dr. Strong related a case of Dysentery attended by an unusual complication. He was called in July to a lady, married, aged 30 years, having dysentery of ordinary severity. The discharges were muco-sanguinolent, not very frequent or profuse. She was treated at first by opiates with the acetate of lead, and subsequently by the saline treatment, (calomel followed by a saline cathartic.) At the end of five days she was convalescent to all appearance, and no fear was entertained of the result. About that time a tumor appeared in the region of the parotid gland, which extended steadily for three or four days until it passed behind the sterno-cleido mastoideus, and downward to the clavicle. This tumor was first noticed as the dysentery seemed better. The tumefaction was of a peculiar stony hardness, feeling like a smooth piece of crockery beneath the skin, which was tense and shining above it. The hardness had no circumscribed border, but gradually subsided into the tissues around. Five or six leeches were applied on the second and third days, as, on the first day, no especial importance was attached to the swelling, which was then trivial. The tincture of iodine was also applied topically. After the tumor was three or four days old, it was poulticed, with a view to suppuration. It soon discharged at the ear a thin, ichorous, purulent matter. On the fifth day it was lanced, and some pus discharged. Shortly before this, Prof. White was called in consultation, and though no alarming symptoms were present, made an unfavorable prognosis on account of having previously seen a fatal result in similar cases. Soon after the tumor was opened, the dysentery recommenced, and the discharges assumed a profuse and bloody character, which was uncontrollable by any means, and in two or three days the patient died.

The opening did not materially diminish the size of the tumor, and before she died a similar action begun upon the opposite side.

Dr. Strong considers the tumor as the cause of the fatal result.

*Dr. White* said that he had witnessed three similar cases. In 1853 he treated a case of dysentery, of a severe grade, by the usual means, viz., anodynes, saline draughts, and the acetate of lead. After a dangerous illness the dysentery disappeared, and the pulse, which had been very frequent, receded to 80. At this juncture a tumor, of peculiar stony hardness, appeared in the left parotid gland, which enlarged so as to press upon the vessels. The surface of the tumor was discolored, and presented a somewhat erysipelatous appearance. The tincture of iodine was applied topically, and the patient's general system supported. No suppuration took place, but the tumor went on enlarging and pressing upon the vessels of the neck, until the patient at last succumbed, without any return of his dysentery, with the pulse at 80 or 90, and to all appearance died from the pressure upon the vessels.

A few days after the death of this case, he met a medical friend in consultation who had seen something similar, and attributed the enlargement to the poisonous effects of lead upon the system.

Previous to this Dr. White had seen two other cases, one, a young girl, in 1849, and another, a child of 7 years, in 1852, who died from a similar tumefaction after dysentery, and without any return of the discharges. Both of these cases took lead. Dr. White inquired whether this was a frequent sequela of dysentery, and whether it was attributable to the exhibition of lead. Lead, more than any other drug, affects the secretion of saliva, checking it, and making the mouth dry.

*Dr. Newman.* Had treated two cases of enlargement of the parotid after cholera, last summer. The first, a young German, when convalescent from cholera had a swelling on the right cheek, over the angle of the jaw, tense, peculiarly hard and shining, and attended by much pain. There was no increase of saliva. Camphorated oil was first applied. Subsequently it was poulticed to suppuration. It was opened and discharged largely. The case recovered, but had dysentery immediately after. He had taken no lead.

The second case was a man who had coma after cholera, but grew better, and then a tumor, like those which have been described, formed in the parotid. At the same time some diarrhœa set in, for which he took two grains of lead every two hours. The tumor was anointed with ung. iodid. and a grain of sulph. morph. rubbed in with each inunction. The tumor was readily discussed and the patient recovered, the exhibition of the lead, and the recession of the tumor going on together.

*Dr. Wyckoff* last summer treated a boy having a mild form of dysentery. He took only two or three pills of opium, grs. ss., calomel, grs. ij.; and after that opium only. Two or three days after his discharge, he returned with a tumor of the submaxillary gland like those which had been described, hard, glistening, with a tense skin. There was no increase of saliva. Directed an ointment of iodid. potass. to be followed by poultices. It recovered rapidly. Dr. W. inquired the general opinion as to the safety of the administration of the acetate of lead.

*Dr. Strong* had used it freely without any unpleasant results, in a variety of complaints. In the case described, he had given it less freely than previously to the same patient.

*Dr. White*, since the cases mentioned had occurred, had avoided it when convenient. He had never given it largely. Preferred, when the indications allowed it, to give the tannic or gallic acids.

*Dr. Newman* then read the following report of a case:

Oct. 27th, 1854. Louis Clauders, a recently arrived French emigrant, was attacked this morning in the street with cholera. He arrived in Quebec about the 20th inst., and had been in this city three days. He was attacked with diarrhœa previous to leaving Quebec, and it had continued, with more or less severity, after his arrival here; and he was in the act of seeking for medical aid when attacked. The first manifestation of the cholera seizure, was a sudden attack of cramps, from which he fell upon the sidewalk.

I saw him a few minutes after he fell, and a careful examination by Dr. Hunt, (who was first upon the ground.) and myself, failed to detect any pulse; and his black, shriveled fingers, and his livid face, proclaimed him already completely collapsed.

I had him conveyed immediately to the Hospital of the Sisters of Charity, and placed under treatment.

Hot bottles were applied to the extremities, and sinapisms to the epigastrium, and along the spine. Calomel, grs. xv., and morphine, gr. j., were administered.

I also directed his head to be shaved, and ice-water kept continually applied.

Beef essence and stimulants to be administered as occasion required, and his condition will admit.

5 o'clock, P. M. It is now about six hours since his admission. A feeble reäction has come on.

His diarrhœa has been very profuse, and the vomiting severe. Neither of these seem to be yet any way controlled; his stomach has rejected medicines, and he vomits whenever he drinks, and his thirst is excessive. The cramps still continue, and are characterized by the attendants as being the most severe of any patient this season.

The pulse can be plainly distinguished at the wrist, and there is a moderate degree of heat of surface, and his hands and fingers are less shriveled, but are yet of a dark, almost mahogany color. His intellect seems unimpaired, and the eyes are very little, if any, injected. There is, however, a peculiar heaving, short respiration, which is frequently observable in these cases, and is to be regarded among the unfavorable symptoms.

I directed a blister to be applied over the region of the stomach, as the mustard had not been sufficient to redden the skin. I also ordered that the cold should be applied more directly to the back of the head and neck. This is to be accomplished by filling a bladder with ice, and pillowing his head upon it. I have prescribed

℞ Prot. chl. hydrg., grs. x.,

S. quinine, grs. x.,

Tannin, ʒss. M. ft. pulv. vi.

One to be given every three hours.

28th, 10 o'clock, A. M. Contrary to what might have reasonably been expected, my patient is still alive, and manifestly improved. The powders sickened him so much that after the administration of two or three, they were abandoned, and as he vomited these as soon as taken, and nothing else substituted was retained, all medication was abandoned, and he may virtually be said to have had no medicine.

He is, notwithstanding, much better than last evening. He has had no diarrhœa since four or five o'clock this morning; and the cramps, which continued the greater part of the night, have now ceased entirely. He still continues to vomit his drinks.

His pulse is increased in strength; the surface is warm; and the dark, congested appearance of the skin has passed away. There is now a slight flush of the cheeks, but no congestion of the eyes. Vesication was produced by the blister.

He is, to-day, to have no medicines, but the ice is to be continued to the back of the head. It should, perhaps, be noticed here, that the irregularity of the breathing has passed away.

5 o'clock, P. M. Continues very slowly, but perceptibly, to improve. The only stimulant he is now taking is wine, which he has asked for, and which his stomach retains in moderate quantities. The cold is yet continued.

29th, 10, A. M. His condition cannot be said to have materially changed since last evening. His bowels have moved off four or five times during the night, but they seem now controlled by astringent enemas given early this morning. His vomiting is more troublesome than his diarrhœa. He has, moreover, become quite intractable and refuses to take food, or medicines, or anything except as his fancy dictates. His chief desire is for water to quench his thirst, which is most intolerable. He is perfectly rational, and converses without difficulty, expressing his disapprobation freely at whatever he dislikes. He has slept at intervals during the night. There is a moderate degree of heat of surface, but he complains of being too warm, and he requires watching to keep him covered.

His pulse is 96, distinct but full. The cold water has been applied irregularly during the night. I have directed

℞ Deut. iod. hydrgr., grs. iij.,
Iodid. potass., grs. x.,
Aqua distil., ℥ss. Ft. sol.

Give five drops every two hours in water. If this does not control the vomiting, to give grain doses of calomel in its stead. The cold applications to be continued to the head.

30th, 11 o'clock, A. M. His pulse is down this morning to 85. He has now had no passage from his bowels for twenty-four hours. An enema given yesterday, of brandy and beef essence, was all retained. His vomiting is very much less. His stomach retains the solution prescribed yesterday, and is less rebellious toward nourishment and drinks. He slept well during the night. His face is still flushed, perhaps a little deepened in color, but his eyes are not injected.

Continue the medication of yesterday. He, to-day, asks for the application of the cold water to his head.

31st, A. M. His pulse is down to 78, and is well-developed. His diarrhœa remains entirely checked. Stimulating and nourishing enemas, which have been frequently given within the last twenty-four hours, are retained He vomits now only occasionally, and the little rejected is of a deep yellow almost brown, color. His thirst is much less. He slept well last night, and is entirely free from pain.

The flush continues upon his cheek, and his eyes, to-day, are somewhat injected.

Directed to-day the more constant application of ice to the mucha, and to give the solution iodo-hydrg. potass., every two hours. If any tendency to stupor appears, to apply cups freely to temples and neck. To sustain him with enemas as before, until he can retain sufficient food upon his stomach.

Nov. 1. Is much better to-day. His pulse is down to 70. His stomach now retains food and drinks. He has vomited but once since my visit of yesterday, and had one movement of his bowels. He to-day requires the use of the catheter, there being retention with a distended bladder.

2d. It has been necessary to use the catheter to-day. He has had several passages from his bowels during the night, and to-day. The discharges are deeply yellow, thin and fœtid. He has been taking tinct. mur. ferri, in thirty drop doses, since last evening, to relieve the stranguary, which has caused him to vomit some.

Directed the reduction of the tinct. M. ferri to gtt. xv doses, and the administration of starch and tannin injections.

3d. About 10 o'clock, A. M., the symptoms of my patient changed suddenly for the worse. At noon he presents the following condition: He is nearly completely comatose, but can be aroused to a sort of semi-consciousness. His face is more deeply flushed, and the eyes more injected than they have previously been. His respiration is but seven a minute, and resembles more a deep drawn sigh than a natural respiration. His pulse is now 100. He has a profuse involuntary diarrhœa, of a deep yellow color, and extremely fœtid. He vomits none. His skin is warm. I introduced the catheter and drew off six or eight ounces of dark colored urine.

I directed him to be cupped freely upon the temples and back of the neck, and cold to be kept continually applied to the head and neck.

℞ Calomel, grs. iij.,
Quinine, gr. ij.

To be given every two hours; and enemas of brandy and tannin to be administered.

7½ o'clock, P. M. Condition not varied from above record at noon. At this visit I have learned, that the blistered surface over the stomach, which has become quite sore, and very painful, was dressed this morning by sprinkling dry morphine over the surface to the amount of two grains, and its absorption may have much to do with the development of the above alarming symptoms. As I had carefully abstained from the use of opiates in any form, or by any method, and the Sister in charge being absent at my visit, I had no information, or cause of suspicion, that they had been thus employed.

4th. The drowsy condition of last night has, in a measure, passed off, but he is still listless, and it requires an effort to arouse his attention, when he will answer questions, and do as he is bidden. His breathing has improved since last evening, but is still slow and sighing. The number of the respirations is now seventeen per minute. His pulse is 88. The diarrhœa is much less. The conjestion of eyes about the same as yesterday, but there is extensive ulceration of the lower half of the right cornea. I introduced the catheter and drew off a very small quantity of dark colored urine.

He complains this morning of his mouth being sore, though there is now redness of the gums, in consequence of which I have withdrawn the calomel.

He is to continue the quinine, and have enemas of brandy and tannin. Cold to be continued to the head and back of neck.

5th. He continued to fail during yesterday, the stupor increasing, and he died last night about 10½ o'clock.

*Remarks.*—The above case illustrates very fully the severity of this complication of cholera. The patient, and the progress of the case, was watched with intense interest by several medical gentlemen. But few patients could be found of a more unpromising aspect than this one when admitted into the hospital. The influence exerted by the cold applications, in producing reäction, I leave others to estimate, merely suggesting that for forty-eight hours nearly after admission, he may be said to have been without any medicines; the medical appliances being all externally employed.

The obstinacy of the vomiting after the checking of the diarrhœa, and the establishment of reäction, is worthy of notice. And this query was strongly forced upon me at the time, from whence, or from what cause proceeds these discharges from the stomach unless it be from the irritation of the brain and nervous centres? Reäction is established, the diarrhœa is checked, the pulse has fallen, the surface is warm and dry; why does he continue to vomit?

The extreme susceptibility of the brain to the influence of opiates is marked in this case; and the extreme danger of employing them in any form, is strongly indicated. I am not prepared, or willing to ascribe, the fatal termination to the influence of the narcotism, for I had from the first, anticipated the possibility and probability of death from cerebral causes. I cannot but regret, however, that the case was complicated by this accidental narcotism of the patient.

*Post-mortem.*—The *rigor mortis* was well marked. Upon removing the calvarium, the blood was found fluid, and large in quantity, the sinuses being crowded with blood. The vessels of the pia mater were abnormally injected, and the surface of the arachnoid was flecked by numerous deposits of caco-plastic lymph lying between the convolutions. The *puncta cruenta* were well marked. No other abnormal conditions were observed in the cerebral cavity. The deposit of lymph indicates the presence of inflammation with sufficient distinctness.

Dr. Wilcox proposed Dr. John G. House as a candidate for membership.

After which the Association adjourned to meet at the office of Dr. White on the first Tuesday in December.

SANFORD B. HUNT, Sec'y.

